# Effect of vibration characteristics and vibror arrangement on the tactile perception of the upper arm in healthy subjects and upper limb amputees

**DOI:** 10.1186/s12984-019-0597-6

**Published:** 2019-11-13

**Authors:** Matthieu Guemann, Sandra Bouvier, Christophe Halgand, Florent Paclet, Leo Borrini, Damien Ricard, Eric Lapeyre, Daniel Cattaert, Aymar de Rugy

**Affiliations:** 1 Team HYBRID; INCIA laboratory, CNRS UMR 5287, University of Bordeaux, 146 rue Leo Saignat, Bordeaux, 33076 France; 2 University Descartes, Paris, France; 3 Departement of Rehabilitation at the Army instruction Hospital, 1 Rue du Lieutenant Raoul Batany, Clamart, 92190 France; 4 Department of Neurology at the Army instruction Hospital, 1 Rue du Lieutenant Raoul Batany, Clamart, 92190 France; 5 Centre for sensorimotor performance HMNS, University of Queensland, Brisbane, Australia

**Keywords:** Amputee, Discrimination, Vibrotactile stimulation, Sensory substitution

## Abstract

**Background:**

Vibrotactile stimulation is a promising venue in the field of prosthetics to retrain sensory feedback deficits following amputation. Discrimination is well established at the forearm level but not at the upper arm level. Moreover, the effects of combining vibration characteristics such as duration and intensity has never been investigated.

**Method:**

We conducted experiments on spatial discrimination (experiment 1) and tactile intensity perception (experiment 2), using 9 combinations of 3 intensities and 3 durations of vibror stimulations device. Those combinations were tested under 4 arrangements with an array of 6 vibrors. In both experiments, linear orientation aligned with the upper arm longitudinal axis were compared to circular orientation on the upper arm circumference. For both orientations, vibrors were placed either with 3cm space between the center of 2 vibrors or proportionally to the length or the circumference of the subject upper arm. Eleven heathy subjects underwent the 2 experiments and 7 amputees (humeral level) participated in the spatial discrimination task with the best arrangement found.

**Results:**

Experiment 1 revealed that circular arrangements elicited better scores than the linear ones. Arrangements with vibrors spaced proportionally elicited better scores (up to 75% correct) than those with 3 cm spacing. Experiment 2, showed that the perceived intensity of the vibration increases with the intensity of the vibrors’ activation, but also with their duration of activation. The 7 patients obtained high scores (up to 91.67% correct) with the circular proportional (CP) arrangement.

**Discussion:**

These results highlight that discrete and short vibrations can be well discriminated by healthy subjects and people with an upper limb amputation. These new characteristics of vibrations have great potential for future sensory substitution application in closed-loop prosthetic control.

## Background

Sensory substitution, the use of a sensory modality to assist or replace another one, is a promising method to restore or compensate sensory loss in a context of amputation. The missing sense can be substituted using stretch, haptic, electric, tactile, visual or auditory feedback [1–5]. Research on sensory substitution has particular interest in the prosthetic domain, and especially for individuals with an upper limb amputation [1, 2, 6–12]. However, one of the biggest issues associated with myoelectric prosthesis control is the absence of efficient sensory feedback. This feedback could enable effective closed-loop control for comparison with actual correction based upon visual feedback loop [8]. The absence of sensory feedback for prosthetic control is highlighted by Peerdeman et al. as one of three main reasons for patients to stop using their prosthesis [13], together with non-intuitive control and insufficient functionality. Because the recovery of the sensory feedback could have a high impact in daily life usage of the prosthesis, this subject is drawing increasing research attention. Using a non-visual feedback signal to control the prosthesis could be advantageous to liberate one’s visual attention which could be directed toward the interaction with the environment, or other tasks.To address this question, sensory substitution has been studied in different contexts looking at substituting grasp force [14, 15], joint position [16, 17], finger force [18], passive touch [19] and hand configurations [3] (see the review of Antfolk et al. for more details [7]). Using the surface of the skin as the interface for sensory substitution has several advantages due to its sensitivity to various stimuli such as temperature, pressure, distortion and vibration [19–22]. In addition, the skin has the ability to transmit both spatial and temporal information. To stimulate the skin, vibrotactile stimulation is commonly used [23–25]. The advantages of such stimuli are the multiple parameters that may be tuned. A vibrotactile stimulation is often characterized by the amplitude and the frequency of vibrations. Other characteristics such as stimulation duration, body localization and intensity of the stimulation may produce signals that could be perceived as distinct [26]. This process is emphasized by the topographic innervation of the skin which provides the element to make the skin an excellent interface for different kind of stimulations. The skin of the arm is innervated by 5 different dermatomes emerging from the spinal roots from C5 to T1. These dermatomes are organized in longitudinal bands around the arm. The roots give birth to cutaneous nerves, which innervate different areas of the arm. These neurological landmarks have been evoked by Cody et al. [27, 28] in their exploration of tactile acuity on different sites in the human upper limb. In this context, accuracy in tactile discrimination is of primary importance. Two studies compared tactile perception of stimulations arranged in longitudinal and transverse orientations in a discrimination task [4, 27]. In the study of Cody et al.[27], the tactile discrimination was explored using a single von Frey hair (rounded tip diameter 0.6mm, rating 150mN at the onset of bending). Better localization acuity was found for the transverse axis. In the study of Witteveen [4], the performance of longitudinal and transversal configurations of vibrors for signaling grasp forces and/or hand aperture by means of vibrotactile stimulation was compared. No significant difference was found between the configurations. However, this study mainly focused on how well people performed the task, but did not provide information about how accurately stimulations were localized. The results of this study completed and confirmed the previous work of Weber and Hamburger on the exploration of tactile stimuli [28, 29]. To our knowledge these studies [4, 28, 29] are the only ones comparing such orientation. The exploration of upperlimb sensory characteristics shows that most of the research has been done at the forearm level [4, 12, 23, 26, 27, 30, 31] and very few at the upper arm level [32, 33]. Based upon principles of sensory physiology and findings of previous studies [4, 28, 29], we presume that vibrotactile stimulations at the upper arm level will be better discriminated when provided circumferentially (in a transversal axis) than linearly (in a longitudinal axis). This hypotheis is based of the fact that the stimulations sent with a circumferential orientation of the vibrors will be more likely to activate nerves endings from various dermatomes compared to stimulations provided linearly which may potentially implicate only one dermatome. We address this question in our experiment where vibrotactile discrimination is tested according to four different arrangements of vibror stimulators, involving two different orientations: a linear orientation aligned with the upper arm longitudinal axis and a circular orientation on the upper arm circumference. Aside from the orientation, the number of textcolorbluevibrors and the space occupied by them are important parameters to consider with the aim to build a set-up that could be integrated into a prosthesis. Previous work reports that the discrimination distance for the upper arm is approximately 3cm [34]. Based on this data, and to test the possible advantage of exploiting the full upper arm surface of subjects, we set two categories of spacings between the center of two vibrors. The first spacing was equal to 3cm and was applied to both orientations. The second spacing was set to be proportional to either the upper arm length or its circumference. This produced inter-vibrors distances longer than 3cm. Combined with the two orientations, these two conditions of spacing created the 4 arrangements tested. In addition to the orientation and spacing between vibrors, the physical characteristics of vibrations may also serve to modulate tactile perception of the signal. The vibrors we chose have been used in numerous studies for their ease of use, small size and low cost [3, 4, 10, 12, 15, 26, 30, 32].

Interestingly, none of these studies have explored the mechanical characteristics of the produced vibrations, such as frequency, intensity, and waveform shape, nor their influence of the perceived signal itself. Instead, these studies directly investigated the capability provided by the vibrotactile signal to identify level of grasping forces [3, 10, 15, 30, 32], or amplitude discrimination [3, 26]. For instance, grasping was feedback either using different locations of vibration on the forearm, or different frequency levels of stimulation [4, 10, 15, 32]. Duration of stimulation could also vary and were often long, from 1.3 sec for Cipriani et al. [26] to as long as the object was held [4]. A specific purpose of our study was to first investigate the influence of some important vibrotactile stimulation parameters on the mechanical characteristics of the vibration produced and on the resulting perception, before using them in a specific task. Among such important stimulation parameters are both the location and the duration of the stimulation [35]. The smallest duration tested so far in studies using the same type of vibror was about 200ms [36]. However, it has been reported that durations longer than 200ms are perceived as bothersome, and that stimulus between 50ms and 200ms are preferred [37]. To produce fast and discrete stimulations and avoid the disadvantages of longer stimulations, we therefore focused on stimulus durations of **60, 100 and 140ms**.To explore and understand the effect of different settings of the stimulation on vibrotactile perception of the skin, we investigated combinations of duration and intensity in two discrimination tasks with the idea that each vibror could convey multiple types of information. The first experiment aimed to evaluate which of 4 arrangements of vibrors elicit the best score in a spatial discrimination task on the upper arm of non-amputee subjects. The second experiment explored how the same combinations of duration and intensity of the stimulation influence the level of perceived intensity of the stimulation, which could be rated as absent (0) weak (1), medium (2) or strong (3). In a second phase, the arrangement that elicited the best scores on healthy subjects was specifically tested for spatial discrimination on 7 participants suffering from an amputation to verify the validity of this arrangement.

## Methods

### Participants

Eleven healthy volunteers (3 women and 8 men; mean age 27.1 ± 7 years [mean ± standard deviation]) participated in the experiment. All but one self-reported to be right handed. None of them had previous experience with vibror stimulation, nor had any known sensory or skin problems. Tactile perception was tested by light touch at different places on the arm. Seven men with an amputation at the humeral level were volunteers to test the best arrangement in the spatial discrimination task. Inclusion criteria was checked by the medical staff. They verified that the stump was fully healed and tested for any sensitivity issues using light touch. All participants signed an informed consent, and the experiment was approved by the national ethics committee (ID RCB 2017-A03609-44).

### Set-up and procedure

#### Vibror setting

Six 10 mm radius vibrors were used (Grove Vibration motor from Seeed company, Shenzhen, China), characterized by a micro motor with an integrated eccentric mass rotating tangentially to the stimulated location (skin) driven by a tension equal to 5 volts. Vibrors were placed too far from joints to activate joint receptors, and the vibrations delivered were too weak to activate proprioceptors from the muscle tendon complex such as Golgi tendon organs and muscle spindles. As such, the vibrations applied here were likely to have only elicited tactile perception from touch receptors in the skin. In this model, frequency and intensity are driven simultaneously, and cannot be adjusted individually. Without any constraint in the circuit, the vibration produced a small but undesired noise, which could potentially disturb subjects and/or give additional auditory information. To avoid this perturbation and elicit different vibration intensities, we added a resistor to the circuit driving the vibror, such that the resistance altered the current driving the vibror. In order to determine the 3 discriminable stimulations, various resistors were previously tested on 3 subjects who did not participate in the main experiment. These intensities included (i) the weakest that allows the subjects to feel the stimulation, (ii) the strongest that could not be heard and that generated no discomfort, and (iii) an intermediate one equally spaced in-between. To determine the resistance levels associated with these 3 intensities, we tested resistors from 120 ohm to 10 ohm. First we started with the highest resistance (120 ohm, eliciting the lowest intensity) and decreased in step of 10 ohm for each subsequent stimulation. We identified the stimulation that could be perceived, and continued to decrease the resistance level (i.e., increase intensity) until the stimulation produced a noise or was felt as uncomfortable. We repeated the procedure in the opposite order starting from the level of resistance that elicited a comfortable stimulation and increasing the level of resistance until the vibration could no longer be felt. With this methodology, we were able to determine an average level for resistances for high and low intensities. The intermediate level was arbitrarily determined as the in-between solution to elicit the medium intensity. As a result, the 3 levels of intensities were achieved using resistors of 80, 50 and 30 Ohm. To create the combination of stimulation, the 3 durations used to elicit brief but detectable stimuli were 60, 100 and 140ms. The vibrors were driven by a Raspberry Pi 3 via a custom-made program written in *Python* *Ⓡ*.

#### Mechanical characteristics of the vibration

**Fig. 1:**
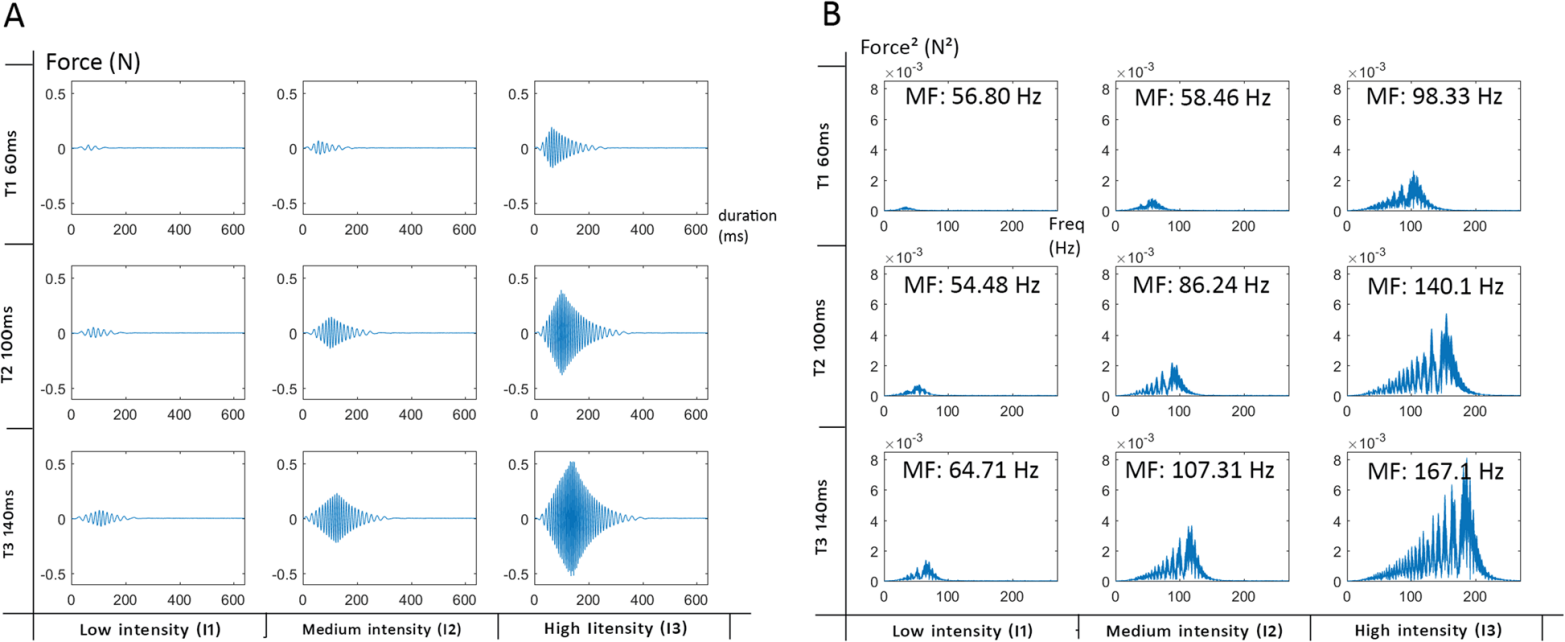
Mechanical properties of vibrations for each combination of time and intensities. **a** represents the force generated (X-direction) by the vibration for each combination of time and intensity; **b** represents the spectre frequency of the stimulation expressed in Hertz on the x-axis and square Newton on the y-axis, Mean Frequency (MF) is labelled for each stimulation

The combinations of stimulation produced using the 3 resistors of 80, 50 and 30 Ohm elicited a current of 62.5, 100 and 167 milliampere (mA), which corresponds to low, medium and high intensity stimulation. These intensities were combined with 3 durations of stimulation of 60, 100 and 140ms. The mechanical properties of the resulting combination of stimulations have been measured with a force transducer (Nano-17, AT I Industrial Automation, Garner, USA) onto which the vibror was firmly taped. Forces along the X direction are displayed Fig. 1. Data were then analyzed with a 5Hz high pass filter because low frequencies produced edge effects with high values for frequencies close to zero. The frequency spectrum (Fig. 1) showed signal variation with a mean frequency (MF) of 56.80 Hz for the weakest signal to 167.1 Hz for the strongest. These stimulation intensities were coherent with the range of Pacinian corpuscle’s sensitivity frequency which is from 40 to 800 Hz as reported by Kaczmarek [38].

#### Vibrors arrangements

**Fig. 2:**
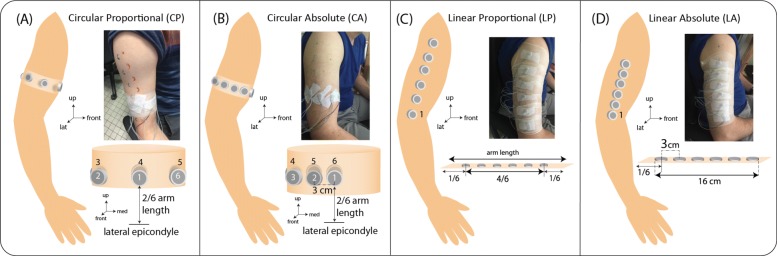
Vibror arrangements tested for each subject. **a** represents the circular proportional arrangement where vibror 1 is placed in front of the arm. The distance between each vibror was proportional to the subject circumference; **b** represents the circular absolute arrangement, where the spacing between center to center of 2 adjacent vibrors is equal to 3cm, and the vibror 1 is at the same place as for the circular proportional arrangement. For (**a**) and (**b**), the vibrors were placed at 1/3rd of the arm length; **c** represents the linear proportional arrangement, where the spacing between 2 vibrors was calculated taking the 4/6th of the arm length between the lateral epicondyle and the acromion, vibror 1 was placed at the bottom and the 6th at the top; **d** represents the linear absolute arrangement, where the center to center distance between 2 consecutive vibrors is equal to 3 cm, with vibror 1 placed at the bottom above the epicondyle

Vibrors were placed on the right upper arm of every subjects for all 4 arrangements tested. They were taped on the subject’s skin and covered with a protective bandage as shown in Fig. 2. The vibror placement referring to each of the 4 arrangements were defined as follows:

- **Circular Proportional (CP)** (Fig. 2a): the vibrors were placed on a circumferential line at 2/6th of the upper arm length between the lateral epicondyle and the acromion. The vibror 1 was placed medially on the biceps, and all vibrors were evenly distributed on the circumferential line such that the distance between the centers of two consecutive vibrors was C/6, with C denoting the circumference of the upper arm. In this arrangement, each vibror was equidistant from its direct neighbors, and the distance between vibror 1 and 6 was the same than the distance between each pair of consecutive neighbors. Inter-vibrors distances for all subjects in the CP condition (IVD-CP) are included in Table 1.

**Table 1:** Participant’s anthropomorphic characteristics

Subject	Gender	Age	4 /6 of upper arm length	upper arm circumf.	IVD-LP	IVD-CP
		(years)	(cm)	(cm)	(cm)	(cm)
S1	Male	21	20	26	4.00	4.33
S2	Male	22	20	29	4.00	4.83
S3	Male	25	23.3	29	4.66	4.83
S4	Female	29	20.7	28	4.14	4.67
S5	Female	22	19.3	25	3.86	4.17
S6	Male	28	20.7	25	4.14	4.17
S7	Male	44	22	30	4.40	5.00
S8	Female	22	17.3	26.5	3.40	4.42
S9	Male	37	21.3	32	4.20	5.33
S10	Male	27	20.7	31	4.14	5.17
S11	Male	21	19.3	28	3.86	4.67
Mean		27.09	20.36	28.14	4.07	4.69
SD		7.04	1.61	2.24	0.32	0.39

- **Circular Absolute (CA)** (Fig. 2b): as for the CP arrangement, the vibrors were placed on a circumferential line at 2/6th of the upper arm length between the lateral epicondyle and the acromion, with vibror 1 placed medially on the biceps. In contrast to CP, however, the distance between the centers of two consecutives vibrors was kept constant to 3cm. As a result, the distance between vibrors 1 and 6 was longer than the other distances, and varied between subjects according to their upper arm circumference as shown in Table 1. With this setting, the length from vibror 1 to 6 occupied a horizontal skin band of 16cm long.

- **Linear Proportional (LP)** (Fig 2c): the 6 vibrors were placed on a line starting from the lateral epicondyle to the acromion. This line was divided into 6 equal parts, such that the first and the last sixth were kept free from vibrors. The 6 vibrors were then evenly distributed on the remaining 4/6th of this line, with vibror 1 placed at the bottom. The distance between the centers of two adjacent vibrors was equal to L/6 where L corresponded to the remaining 4/6th of the subjects’ upper arm length. Inter-vibrors distances for all subjects in the LP condition (IVD-LP) are included in Table 1.

-**Linear Absolute (LA)** (Fig. 2d): as for the LP arrangement, the vibrors were placed on the vertical line between the lateral epicondyle and the acromion. The first vibror was placed at the bottom as it was for the LP arrangement. In contrast to the LP arrangement, the distance between the centers of two consecutive vibrors was maintained at 3cm. As for the CA arrangement, the 6 vibrors occupied a skin band of 16cm long and 1cm width, but in a vertical orientation.

#### Procedure with healthy subjects

All healthy subjects participated in the 2 experiments where the 4 arrangements were compared. The spatial recognition task (experiment 1) was tested first, with 2 to 3 min rest between each arrangement, followed by the intensity perception task (experiment 2). For both experiments, the procedure was organized in the same way.

*Spatial discrimination tas*k: First, a vibror arrangement was randomly assigned. Then, a familiarization phase occurred. The position of each vibror was learnt following a procedure of three runs of the vibror 1 to 6 with 500ms of stimulation duration and 500ms intervals without any stimulation (pause). The stimulation was done at the maximum intensity (167mA). Then, three repetitions in the opposite direction (vibror 6 to 1) were realized. This procedure was repeated with stimulation duration and intervals without stimulation of 200ms each and 100ms each, respectively. During this sequence, the experimenter verbally indicated which vibror was activated by saying its number. After this familiarization phase, the spatial discrimination task began. A block of 162 stimulations was delivered to each participant for each of the 4 arrangements tested. Each block includes 3 repetitions of a stimulation for each of the 9 combinations of duration and intensity, for each of the 6 locations of vibrors. The order of stimulations was randomized within each block before being send to each participant. After each stimulation, the experimenter asked the participant which vibror was activated. The subject had to answer the location of the stimulated vibror between 1 and 6. At the end of the 162 stimulations, success rate was calculated, and the next arrangement to be tested was assigned.

*Perceived intensity task*: This task proceeded in the same manner as the spatial recognition task. The order of arrangements, as well as the order of the 162 stimulations within each block per arrangement, were similarly randomized. The familiarization occurred similarly except that instead of the location of stimulated vibror, the experimenter mentioned the level of stimulation intensity applied between 1(weak), 2 (medium) or 3 (strong). During the test phase, participants had to indicate after each stimulation how strong he/she felt the stimulation (possible answers: 0 = no feeling, 1 = weak, 2 = medium and 3 = strong intensity).

#### Procedure with patients

**Fig. 3:**
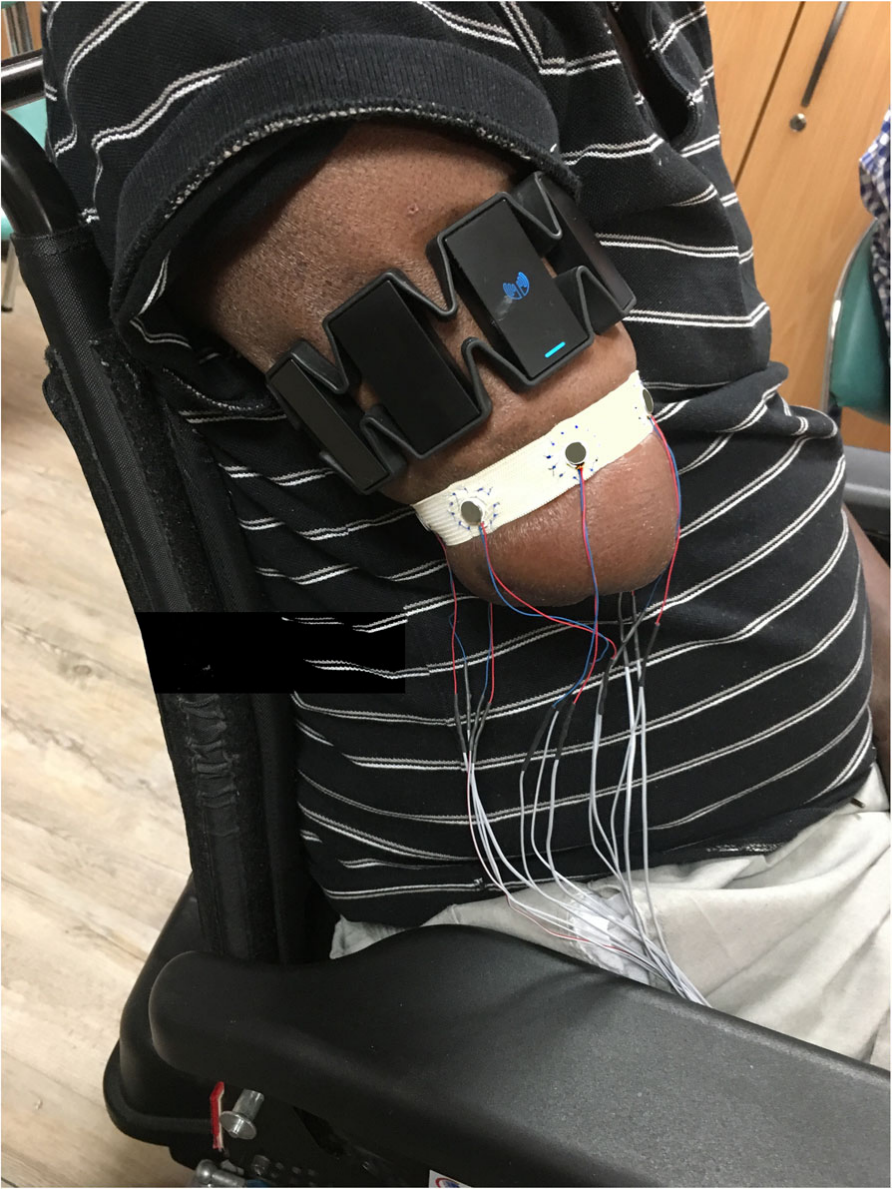
Confusion matrix representation of correct answers for the 7 patients testing the CP arrangement in the spatial discrimination task. Patients undewent two sessions. For both matrices, X axis represents the stimulation sent as “order” and the Y axis the patient’s answer. The gradient color corresponds to the recognition rate for each of these vibrors combinations. Darker color represents a higher recognition rate for the answered vibror. Correct answers correspond to the diagonal, for which the answered vibrors corresponded to the stimulated ones. Errors occurred whenever the answered number differed from the stimulated one

In a second phase, the arrangement that elicited the best scores on healthy subjects was specifically tested for spatial discrimination on 7 participants suffering from an amputation to verify the validity of this arrangement. In addition, the length of the stumps was often insufficient to test longitudinal arrangements. The 6 vibrors were placed circumferentially on the stump and activated with the lowest resistance (30 Ohm) eliciting the highest stimulation intensity used in the main experiment Fig. 3. This was designed to ensure that each vibror was well perceived, as the main focus of this experiment on patients was on the localization aspect rather that on the perceived level of intensity. We quickly cheked with each amputee whether all vibrors were well perceived. In cases a vibror was less perceived than the others, the resistance was decreased by a step of 10 Ohm to further increase the intensity of the stimulation to that specific vibror. In practice, this only occured on aproximately 10% of cases and might be attributed to healing process and scar tissue at the level of the stump. We used the intermediate duration of stimulation employed in the main experiment (100ms). In this experiment, we only focused on spatial discrimination to evaluate the patients’ capacities. The familiarization phase was then started following the procedure previously described. During this sequence, the experimenter verbally indicated which vibror was activated by saying its number. This familiarization phase took about two minutes. Patients started the discrimination test right after the familiarization phase. The first test block consisted of spatially locating across 24 stimulations organized in 4 repetitions of 6 stimulations for each of the 6 vibror’s location, presented in a randomized order. This test was repeated in a second block to evaluate the consistency of the answers and if a learning effect was present.

### Data Analysis

Descriptive statistical analysis were calculated for anthropomorphic characteristics of participants. Both experiments were analyzed using a Generalized Linear Mixed Model (GLIMMIX method). Effect of each factor (arrangement, duration and intensity) was calculated and two by two comparison was conducted for each category within each factor. For the first experiment (spatial recognition), the outcome was binomial: the participants’ identification of the stimulated vibror was either correct or not. The statistical analysis calculated the probability of correct answers in relation to the studied factor.

For the second experiment (perceived intensity), the outcome variable was ordinal (4 levels Likert scale for the 4 level of intensity). The multimodality character of this dependent variable was specified to the GLIMMIX model analysis. The effect of each factor (intensity, duration and arrangement) was calculated. Two by two comparison within a same factor was made on the odds of having an answer higher (up of 1 level) between the compared categories for one factor adjusted to the others (i.e. likelihood of having a higher response with duration 100 ms compared to duration 60 ms adjusted to intensity and arrangement).

Two by two comparisons are expressed in odds ratio with a 95% confidence interval. Statistical level of significance was placed at 0.05. All statistics were performed using SAS Studio Basic Edition version 3.7.

For the spatial discrimination test realized with patients, data were analyzed as binary values: 1 for correct localization and 0 for incorrect localization. It was then reported as percentage (Table 5). A Wilcoxon signed-rank test was used to evaluated if a change occurred between the first and the second session.

## Results

### Anthropomorphic characteristics

The mean (±SD) ot the 4/6th lengths and circumferences of the upper arms of the 11 subjects were 20.36 (±1.6) and 28.14 (±2.2) cm, respectively (Table 1). The available lengths of all healthy subjects were sufficient to place the 6 vibrors with a minimum spacing of 3cm between the center of 2 adjacent vibrors for the arrangements with an absolute spacing (Fig. 2b and d). For the arrangements with a proportional spacing (Fig. 2a and c), the distances between the center of two consecutives vibrors were 4.07 (±0.32) cm for the LP arrangement and 4.69 (±0.39) cm for the CP arrangement. Paired t-test shows a significant difference (p <0.001) between the inter-vibrors distances (IVD) obtained for the CP and LP arrangement, with larger distances for CP.

Patients’ characteristics are presented in Table 2. The etiology of all amputations was traumatic. Overall, upper arm circumference was smaller than healthy subjects but allowed a space between the center of two adjacent vibrors of more than 3cm. The stump was too short to correctly place the vibror. For the longer one (26cm frome the base of the stump to the acromnion), if we remove the 5cm to not cross the shoulder, it remains 21cm minus the 6cm (1cm of diameter for each vibror) and divided by 5 spacings we obtain 3cm. All the other stumps had lower lenghts so, the arrangements with linear linear orientation of the vibrors can not be used for them. Time since amputation was noted to be variable but did not appear to affect task performance.

**Table 2:** Patient’s anthropomorphic characteristics

Subject	Upper arm circumference	Stump lenght	Side of the	Cause of the	Age	Time since
	(cm)	(cm)	amputation	amputation	(years)	amputation (years)
P1	23	23	Right	Traumatic	59	<1
P2	24	21	Right	Traumatic	57	40
P3	19	26	Left	Traumatic	34	9
P4	20	14	Left	Traumatic	67	43
P5	18	25	Left	Traumatic	58	36
P6	30	20	Right	Traumatic	48	3
P7	20	25	Left	Traumatic	71	24

### Experiment 1: spatial recognition

**Fig. 4:**
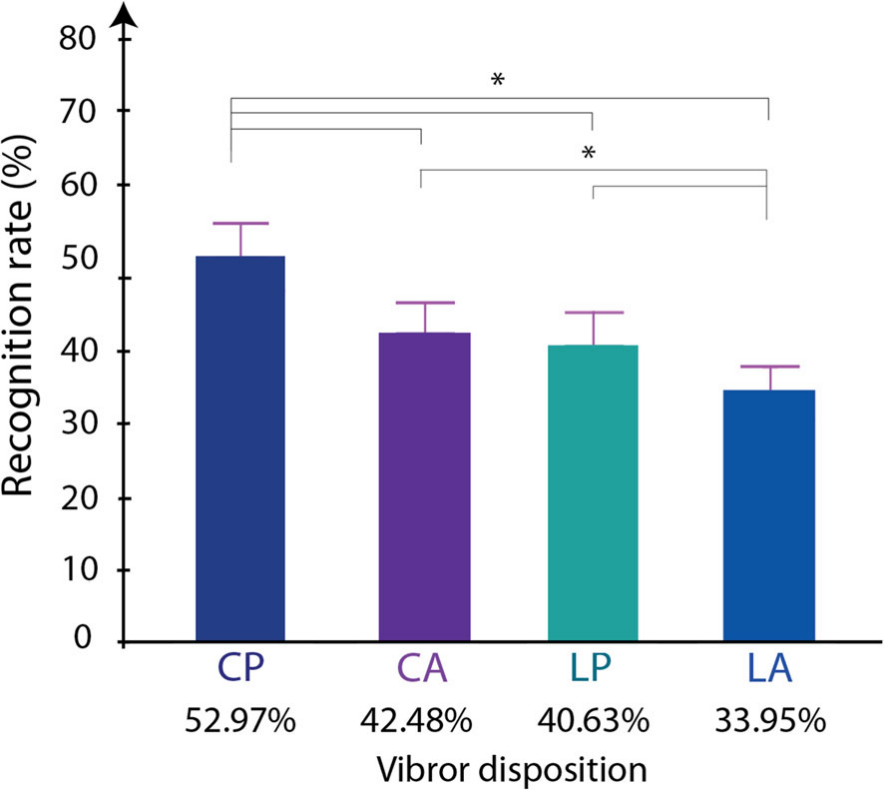
Comparison of success rate percentages for each of the four vibrors arrangement, CP: circular proportional, CA: circular absolute, LP: linear proportional, LA: linear absolute

**Effect of vibror arrangement, stimulation duration, and stimulation intensity on spatial recognition success rate.** The first experiment was conducted to compare the capacity of the participants to correctly identify the vibror position between the 4 different vibror arrangements, and explore the effect of stimulation duration and intensity of performances. Overall, the results reported in Table 3 indicate that the circular proportional arrangement enables a much better spatial recognition than all other arrangements, and that stimulation duration and intensity greatly affect this capacity. The statistical analysis showed significant effects of the arrangement (F = 50.29, p <0.0001), of stimulation duration (F = 89.64, p <0.0001) and intensity (F = 322.20, p <0.0001). Two by two comparison between arrangements showed a significant difference in favor of CP compared to each of the other arrangements. The probability of correctly answering with the CP arrangement was 1.63 times higher than with the CA arrangement (p <0.0001), 1.78 times higher than with the LP arrangement (p <0.0001) and 2.52 times higher than with the LA arrangement(p <0.0001) (Fig. 4). The LA arrangement had the lowest probability of correctly answering (p <0.0001) compared to all of the other arrangements. Only LP combined with CA did not differ (p = 0.23).

**Table 3:** Two by two comparison with probability of correct answer in the spatial discrimination task for each factor

Comparisons	OR	CI95%		*p* value
Disposition CP/CA	1.63	1.42	1.88	<.0001
Disposition CA/LA	1.54	1.33	1.79	<.0001
Disposition CA/LP	1.10	0.95	1.26	0.23
Disposition CP/LA	2.52	2.17	2.93	<.0001
Disposition CP/LP	1.78	1.55	2.06	<.0001
Disposition LA/LP	1.41	1.22	1.64	<.0001
Time 140 vs Time 60	2.39	2.10	2.72	<.0001
Time 140 vs Time 100	1.43	1.27	1.63	<.0001
Time 100 vs 60	1.66	1.46	1.89	<.0001
Intensity High vs Low	5.41	4.73	6.19	<.0001
Intensity High vs Medium	1.47	1.31	1.66	<.0001
Intensity Medium vs Low	3.67	3.22	4.19	<.0001

Probabilities are expressed in odds ratio (OR) and confidence interval (CI 95%)

*Note:* LP refers to lingitudinal proportional disposition, LA to longitudinal absolute, CP to circular proportional and LA to circular absolute

As the higher score obtained for CP than for LP could be explained by higher inter-vibrors distances for CP, we further assess the potential correlation between inter-vibrors distances and success rates within both CP and LP arrangements. For both arrangements, the correlation coefficient was not significant (CP: r = 0.33, p = 0.31 and LP: r = -0.04, p = 0.89). Therefore, despite the fact that best scores were obtained for the arrangement that involved the higher spacing (CP), success rate could not be correlated to individual inter-vibrors distances within each condition.

The effect of time and stimulation intensity was also reported and analyzed in two by two comparisons. The probability of obtaining a correct answer was higher when the stimulation duration increased: 2.39 and 1.43 times higher for 140ms compared to 60ms and 100ms, respectively, and 1.66 times higher for 100ms compared to 60ms, all p <0.001. The probability of success also increased with the stimulation intensity: 5.41 and 1.47 times higher for high intensity (I3) compared to medium (I2) and low intensities (I1), respectively, and 3.67 times higher for medium compared to low intensity, all p <0.001).

**Fig. 5:**
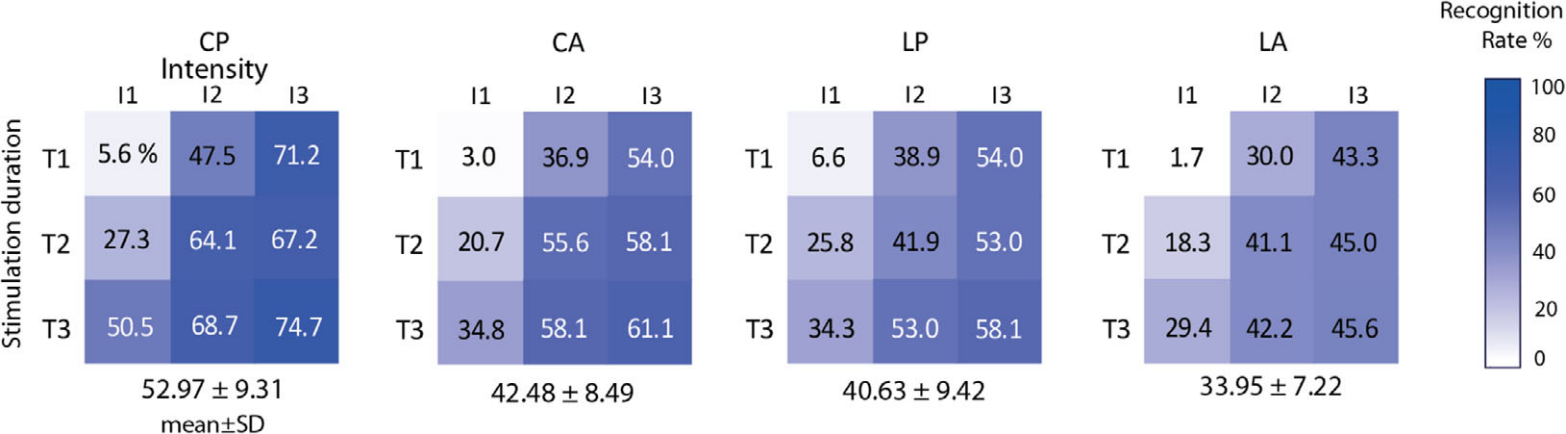
Mean success rate (in percentage) obtained per combination of stimulation for the 4 vibrors arrangements (CP, CA, LP, LA). Combination of stimulation are characterized by time: T1 = 60ms, T2 = 100ms and T3 = 140ms and intensities: I1 weak = 62.7mA, I2 medium = 100mA and I3 strong = 167 mA

Figure 5 illustrates the success rate obtained for the 9 combinations of stimulation for each of the vibror arrangements tested. Mean results indicate that the best score obtained in the CP arrangement (53%) was substantially higher than the second-best score obtained in the CA arrangement (42%), which is close to that obtained in the LP arrangement (40%), and higher than that obtained in the LA arrangement (34%) (Fig. 4). For all vibror arrangements, the worst score was obtained with the combination involving the shortest duration and the weakest stimulation intensity (T1-I1), and the best score was obtained for the combination with the longest duration and highest intensity (T3-I3). For a given stimulation duration (horizontal lines) when the intensity increased, the recognition rate increased too. The same behavior was observed for a given intensity (vertical lines), with recognition rates that increased with stimulation duration. For the CP arrangement, 6 of 9 combinations had a recognition rate higher than 50% (Fig. 5), which is relatively good as the probability of having a correct answer by chance was 16.67% (1/6).

**Fig. 6:**
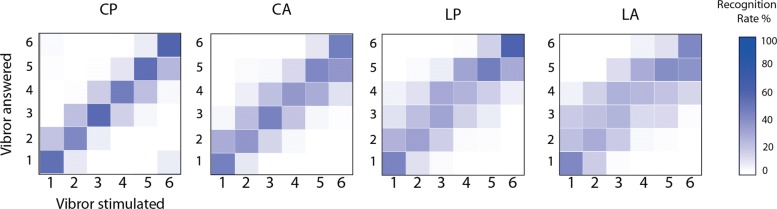
Confusion matrix representation of correct answer for the 4 vibror arrangements. The X-axis represents the stimulated vibrors, the Y-axis the vibrors answered by the participants as being vibrated. The gradient color corresponds to the recognition rate for each of these vibrors combinations. Darker colors represent a higher recognition rate for the answered vibror, while lighter colors represent a weaker. Correct answers correspond to the diagonal, for which the answered vibrors corresponded to the stimulated ones. Errors occurred whenever an answered vibror number differed from the stimulated one (i.e., whenever out of the diagonal)

**Spread of errors.** To visualize the distributions of correct and incorrect answers, confusion matrices were created for the 4 vibror arrangements in Fig. 6. A color gradient was used to represent the recognition rate associated with each answered vibror (Y-axis) as a function of the vibror that was actually stimulated (X-axis). In this representation, darker colors represent higher response rates, such that a dark diagonal indicates correct responses (i.e., stimulated vibrors recognized as such), whereas a colored area spread around the diagonal indicates confusions with neighbor vibrors. It is clear from Fig. 6 that the Circular Proportional arrangement (CP) provided the least confusion with the darker diagonal representing correct answers. For the CA and LP arrangements, the diagonals are less pronounced, with more frequent errors. This spread of errors was even more apparent for the LA arrangement, where the stimulation was sometimes perceived at 2 or 3 vibrors away from the stimulated vibror.

### Experiment 2: level of tactile perception of the stimulation analysis

**Fig. 7:**
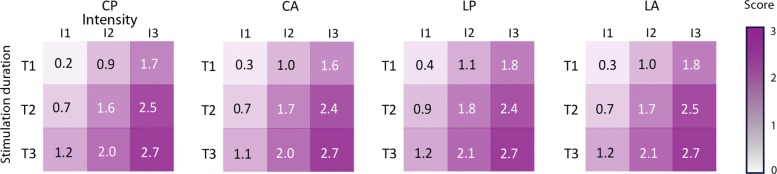
Representation of the perceived stimulation for all configurations. Each cell represents the mean score obtained for a given combination of duration (Y axis: T1 = 60ms, T2 = 100ms and T3 = 140ms) and Intensity (X-axis: I1 weak = 62.7mA, I2 medium = 100mA and I3 strong = 167 mA). Scores range from 0 (absence of perception) to 3 (maximum perceived intensity)

The second experiment was conducted to analyze how strong the participants perceived the 9 combinations of duration and stimulation intensity on a scale from 0 to 3. The mechanical characteristics presented in the “Methods” section showed that both the magnitude and frequency content of the vibration delivered is greatly affected by variation of stimulation characteristics which are duration and intensity. With this experiment, we wanted to analyze the impact of these variations on the perceived intensity of the vibration by subjects. The statistical analysis revealed significant effects of the arrangement (F = 14.67, p <0.0001), of the duration of stimulation (F = 1122.33, p <0.0001) and of the intensity of stimulation (F = 1784.66, p <0.0001). Table 4 presents the two by two comparisons on perceived intensities for each factor. An odd ratio (OR) superior to 1 means that the stimulation is perceived stronger than the condition to which it is compared. For each of the 3 comparisons, level of intensity has an effect on the tactile perception with a higher probability of feeling the stimulation stronger (answer with a higher level) for the higher levels of intensity (all p <0.0001). This is not surprising as this coincides with the magnitude and frequency of the vibration that were found to greatly increase with the intensity of the vibror activation (Fig. 1). More interestingly, stimulation duration has also an effect on the perceived level of intensity. The probability of feeling the vibration stronger is systematically associated with longer durations (all p <0.0001). This could also be explained by the magnitude and frequency of stimulation that were found to both increase with the duration of vibror activation (Fig. 1). The last effect highlighted by the analysis concerns the arrangement. Results from Table 4 indicate that arrangements with linear orientation of vibrors induce a stronger feeling of the stimulation for LP and LA when compared to CA or CP. However, this effect is associated with small effect size given by the odds ratios (between 1.20 and 1.50).After showing the effect of the different factors in Table 4, we analyzed how the answers were distributed for all 9 combinations of stimulation for each of the four arrangements (Fig. 7). The results presented in Fig. 7 appear to mirror the changes in magnitude and frequency of the vibration elicited by the different combination of intensity and duration of vibror activations (Fig. 1). Each cell of the matrices corresponds to the mean score answered for a combination of duration and intensity of stimulation. A score of 0 corresponds to an unperceived vibration, and a score of 3 represents the maximum perceived intensity. For a given intensity (vertical lines), stimulations were felt stronger when durations increased. For a given duration (horizontal lines), stimulations were felt stronger when intensity increased. The poor scores (close to 0) obtained for all arrangements using the combination T1-I1, revealed that participants could barely perceive the stimulation. In addition, it is important to note that different combinations of stimulation parameters were able to elicit close to similar perceived intensities. For instance, for all configurations, the combinations T1-I3 and T2-I2 elicited scores of perceived intensities between 1.6 and 1.8. The same proximity of scores was observed for the combinations T1-I2 and T3-I1 (0.9 to 1.2). This can be explained by the mechanical wave produced with those combinations (Fig. 1). Combinations of stimulation parameters that elicited close to similar perceived intensities also elicited very similar magnitude and frequency of vibrations: T3-I1 = 64.71 Hz ; T1-I2 = 58.46 Hz and T1-I3 = 98.33 Hz ; T2-I2 = 86.24Hz.

**Table 4:** Estimated probabilities of perceiving the stimulation intensity stronger across multiple variables

Comparisons	OR	CI 95%		*p* value
Disposition CA/CP	1.01	0.87	1.16	0.91
Disposition LA/CA	1.23	1.07	1.42	0.003
Disposition LP/CA	1.49	1.30	1.72	<.0001
Disposition LA/CP	1.24	2.17	2.93	<.0001
Disposition LP/CP	1.21	1.05	1.39	0.0007
Time 140 vs Time 60	38.32	32.92	44.61	<.0001
Time 140 vs Time 100	4.03	3.55	4.57	<.0001
Time 100 vs Time 60	9.52	8.34	10.85	<.0001
Intensity High vs Low	293.47	243.55	353.62	<.0001
Intensity High vs Medium	13.10	11.44	15.01	<.0001
Intensity Medium vs Low	22.40	19.36	25.92	<.0001

Probabilities are expressed in odds ratio (OR) and confidence interval (CI 95%)

Note: LP refers to longitudinal proportional disposition, LA to longitudinal absolute, CP to circular proportional and LA to circular absolute.

**Table 5:** Patients scores at the discrimination task

Subject	Session 1	Session 2
S1	66.70	83.33
S2	83.33	87.50
S3	62.50	91.67
S4	58.33	83.33
S5	78.17	83.33
S6	62.50	66.67
S7	62.50	87.50
Mean	67.72	83.33
SD	9.34	7.98

### Spatial discrimination with patients

**Fig. 8:**
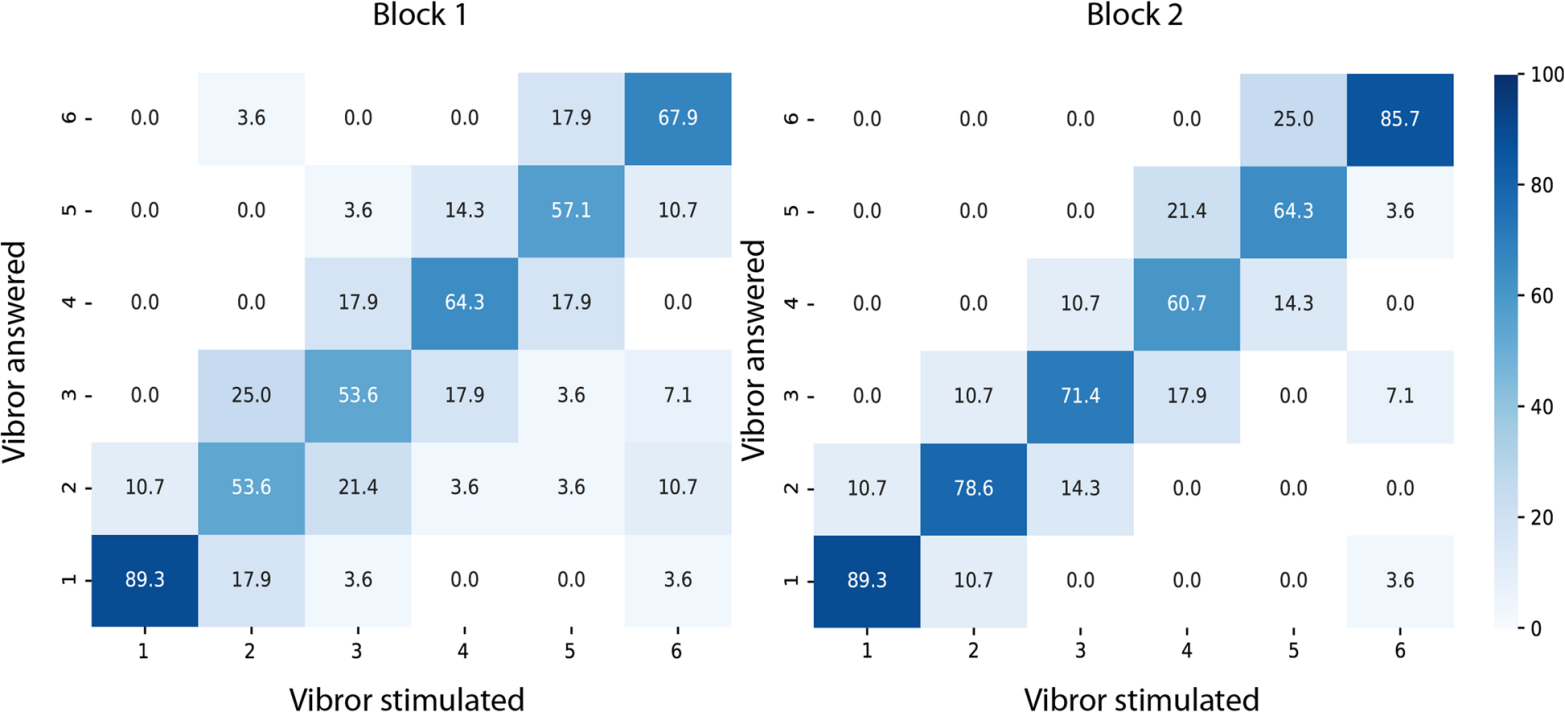
Example of one patient with vibrors encapsulated in a plastic piece and attached to an elastic band by surgical file

Results of correct recognition rate obtained with the 7 patients are shown in Table 5. The arrangement of vibrors used was the circular proportional (CP). Considering the short learning phase, results obtained are consistent with the means and best scores obtained with healthy subjects for the combinations T2 (100ms) and the intensity 2 and 3 which were about 65 to 70% (Fig. 5). Each patient underwent 2 consecutives discrimination tests with 2 minutes rest in between. The Wilcoxon signed-rank test between session 1 and 2 presents a significant difference (p <0.05) that demonstrates a real progression between the sessions. Confusion matrices reporting all answers for session 1 and session 2 are presented in Fig. 8. The matrix of the first session shows a wider spread of errors around the stimulated vibrors which is reduced to adjacent vibrors in the matrix of the session 2. These results confirmed the effectiveness of the CP arrangement and validated its accuracy on a sample of people with humeral amputation. The better scores obtained for vibror 1 and 6 compare to the others might be due to the familiarization phase, were vibror 1 and 6 are always the first and last vibror to be stimulated. They might have been used as reference points to learn the positions of all vibrors.

## Discussion

The main objective of this study was to estimate the effect of vibration characteristics and arrangement of vibrors on tactile perception in healthy subjects and amputees. Vibrations characteristics were varied by combining different durations and intensities The results obtained on spatial accuity and intensity perception have important applications for future integration of sensory stimulation systems in closed-loop myoelectric control.

### Influence of the arrangement

The circular proportional arrangement (CP) obtained the best score for the spatial discrimination task (53%). This result was significantly different compared to the 3 other arrangements with less errors spread (Fig. 6). When the participants misidentified the stimulated vibrors, their answer usually involved a vibror adjacent to the correct response. The same pattern was found with the patients (Fig. 8). The Circular absolute (CA) and longitudinal proportional (LP) arrangements had lower scores with no statistical difference between them. The longitudinal absolute arrangement (LA) placed last, with the worse discrimination score. Those results conpleted the observations of Cody et al. [27], where the tactile localizing acuity was greater in the transversal axis than in the longitudinal one at the forearm level, for the upper arm level. The observed variations in tactile localization might be attributable to peripheral innervation patterns [28]. This proposition matches with the configuration of the dermatomes at the arm level. The arrangement with a circular orientation of vibrors maximized the chances to provide stimulations to all 5 dermatomes of this region which are C5; C6; C7; C8 and T1, whereas the linear orientation of vibrors is likely to provide stimulations on a same and unique dermatome. As the dermatomes are innervated by cutaneous nerves emerging from roots localized at different levels of the spinal cord, their stimulation should have induced an integration at different levels that probably helped localization when compared to stimulation of the same dermatome. In addition, better results obtained for circular orientations compared to longitudinal ones might be explained by the morphological description of this primary afferent receptive field which is typically oval-shaped and oriented in the longitudinal axis [39]. Regarding this description, stimulations on a transversal axis are more likely to elicit different mechanoreceptive units than in a longitudinal axis.

The distance between two adjacent vibrors also appears to be an important element in tactile discrimination. For the arrangement with proportional spacings, the distances between the centers of 2 vibrors were always larger than the absolute ones with a mean spacing of 4.7 cm and 4.1 cm for the CP and LP respectively. In these conditions, the arrangements always obtained better scores when compared to the absolute ones (CP vs CA and LP vs LA). The results obtained with larger spacings can be explained by the large receptive fields of primary afferent nerves of the Pacinian receptors (100mm2) at the arm level [25]. Larger spacings might be likely to stimulate different mechanoreceptors compared to smaller ones [28]. In this context, the scores observed between CP and LP arrangement could be partly due to higher inter-vibrors distances obtained for CP compared to LP. Please note that this differences in spacing were justified by our intention to maximize the distances for each condition as a function of participant’s morphology. However, higher scores obtained for CA than for LA, despite similar inter-vibrors distances strongly suggest that spacing is not the only factor that favored the circular orientation. Surprisingly, the results obtained by patients in the spatial discrimination task showed that even for a distance between the center of two adjacent vibrors just over 3 cm, the vibror arrangement was large enough to get scores over 75% of recognition rate (Table 5).

### Influence of intensity and duration

Stimulation intensity had an important effect on the likelihood to answer correctly (Table 3). When the intensity increased, the probability of giving a correct answer increased too. As the vibration force generated by the stimulation is stronger with higher intensity (Fig. 1), the skin deformation and the amplitude of the wave produced is bigger as well. The combination of stimulations with a same duration but different levels of intensity were perceived as different signals (Fig. 7). This pattern is similar for all arrangements and for the 3 durations. It confirms that intensity is a good candidate for signal modulation. These results are coherent with our expectations and the mechanical properties of the vibration. However, the weakest intensity made the discrimination difficult for participants with a best score less than 7% of correct answers for the combination involving the shortest duration (Fig. 5). These scores were probably due to the perception threshold that might not have been reached for all participants, and when the stimulation was felt, it was probably too weak to enable identification of the position between the 6 possible locations.

The stimulation durations used in our experiment were 60, 100 and 140ms. The physical properties described in the introduction and in Fig. 1 show that the mechanical signal did not reach its steady state for any durations with a signal still in its raising phase. The results obtained in both experiments demonstrated that longer the duration, better the recognition and that the 3 durations induced different levels of tactile perception of the signal. For a same intensity (vertical lines in Fig. 7) the intensity of the stimulation was perceived stronger for longer duration stimulations than for shorter ones. This effect was highly visible for the combinations involving the 60ms duration and the medium and high intensities (I2 and I3). For all the combinations involving I2 (100mA) the perception of intensity doubled (score 1 to 2) when duration increased (from 60ms to 140ms). If the duration was not to influence tactile perception, participant’s answers should have been closer to 2. These findings highlight that independently of the arrangements, the scores obtained in the discrimination task increased with both stimulation intensity and duration (Fig. 5) and showed how duration and intensity played a role in the production of different sensory signals. The lack of steady state and the hardly perceived stimulation induced by short duration might encourage the use of longer durations. However, duration over 200ms are known as bothersome [37], and could not serve our purpose which is to build a closed-loop feedback system to enhance online movement control of prosthesis. Using vibrotactile signal to indicate the orientation of the elbow, the signal not only has to be noticeable, but, should also be as short as possible not to induce detrimental delays in the feedback control loops.

### Advantages of using 6 vibrors

The choice to use 6 vibrors relied on the task objective, the space occupied by the set-up and possible prosthesis integration. The two points discrimination described in the literature for the arm is around 3 and 4 cm [24, 40, 41], we used this data as a baseline for the set-up arrangement. Having 6 vibrors allowed us to implement the 4 arrangements respecting this spacing without crossing articulations (for the arrangement with linear orientation) or other vibrors (for the circular ones). From a practical point of view, having 6 vibrors around the upper arm as a bracelet appears to be more convenient and potentially more suitable than a longitudinal band for prosthesis integration. The width of the ribbon corresponds to the diameter of the vibror which is equal to 1cm. In addition, the level of amputation was different between patients, but the ribbon of vibrors around the remaining stump fitted perfectly for all patients (Fig. 3) whereas the linear arrangement needs a minimum length which is larger than the stump size for most patients. With such an arrangement of 6 vibrors, we are currently using the present results to inform the design of a closed-loop artificial elbow control with sensory substitution. In this context, the association of each vibror with a particular range of 20 deg at the elbow joint would enable coding for 120 deg amplitude movements, which corresponds approximately to the useful range of motion for the elbow. As both the mechanical characteristics of the vibrations and the perceived intensities were found here to greatly vary with duration, discrete stimulation of fixed duration appears necessary to elicit consistent perception. And as mentioned earlier, the stimulation should also be as short as possible not to induce detrimental delays in the continuous feedback control loops. As the present results indicate that good spatial localization is obtained with 100ms stimulation, we are currently testing closed-loop myoelectric elbow control with on-off sequences of 100ms stimulations of the vibror that corresponds to the ongoing location of the elbow. While preliminary results obtained with this setting are encouraging, fine tuning in relation to the specific task and control mode constitutes important work in progress.

### Drawbacks of our study

The main drawbacks of our work were the relatively poor success rates obtained in the spatial discrimination task with healthy subjects who explored all stimulations combinations. The best mean success rate was 53% for the CP arrangement with some combinations reaching up to 75%. These results might appear weak compared to other studies where success rates in a discrimination task can reach 80% [2, 42, 43]. This can be explained by methodological differences. In our study, participants had to recognize the active vibror between 6 possible locations. The chance probability of a correct answer was 16.67%. This is relatively low compared to studies using a forced choice design with two choices. The probability of correctly answering by chance is, in that case, 50% [36]. Another explanation comes from the scores obtained from stimulation combinations involving the shorter duration and the smallest intensity (T1: 60ms, I1: 62.5mA). These stimulations were often too weak to reach the perception threshold. Without this condition, the success rate for the CP arrangement increases to 60% of correct answers. These findings were also influenced by the choice of a fixed level of stimulation without adjusting it for each participant. As each individual has their own level of tactile perception, due to the thickness of their skin and other physiological characteristics, a subject-specific calibration could have been a better option. However, our goal was to characterize the effect of duration and intensity on the vibration themselves first, before observing their impact on the discrimination tasks. In this context, having a set of fixed duration and intensity of vibror stimulations was the price to pay to have a clean comparison of the influence of these parameters on the physical properties of the vibration produced. This choice may have been responsible for inter-individual differences in tactile perception, but should not have affected the overall conclusions drawn about spatial arrangement of vibrors and the role of stimulus duration.

The familiarization phase might also have influenced the results. In our experiments, none of the participants had previously experienced vibrotactile stimulations and the familiarization phase was really short. For the spatial discrimination task subject underwent 3 repetitions of activations of the 6 vibrors in each direction (1 to 6 and 6 to 1) at one intensity and different durations. For the intensity perception task it was the stimulation of 3 different intensities and one duration. This phase was too short to allow any real learning between stimulations and vibrors’ position. The changes in the experiment with patients shows they were still in a learning phase with their scores increasing between each session. In addition, no feedback was given to the participant neither after each stimulation nor at the end of a block. Doing so, we wanted to explore the natural and intuitive character of our stimulation and see if, with a minimum of training, the participant could answer well. Our results reflect therefore the behavior of naïve participants and patients upon their very first exposure to this kind of stimulation. This characteristic can be seen as an advantage for patients with an amputation, for whom learning associated with myoelectric control already requires a long time. Furthermore, we are confident that adding a short learning phase could substantially improve our results when referring to the work of Stronks et al. [44] who showed that 20 minutes of training is enough to increase intensity discrimination and spatial acuity.

The absence of woman in the patient’s group might have also affected our results. The literature describes differences in skin composition between male and female [45, 46]. The men skin is 1.2 times thicker than the women skin; men produce more sweat (1.7 times) than women and are less sensitive to pain and temperature [46]. All those factors may contribute to changes in vibrotactile perception. Also, Woodward et al. show than skin compliance was different between genders as it was lower for male than for female [45]. However, they did npt find that gender was correlated to 2 points discrimination thresholds. It remains that the arm circumference of women is more likely to be smaller than that of men, which might induce smaller spacing between vibrors for women. Gender differences should therefore be further investigated and include in the future.

### Conclusion and perspectives

The results of our experiments are encouraging and provide new information about reaction to vibrotactile stimulations combining intensities and durations. During the development of our experiment, we kept in mind the target population of patients for which this system is dedicated: people with upper limb loss, and more specifically at the humeral level. For this type of amputation, the prostheses socket encompasses the entire remaining arm to finish over the shoulder. The socket appears to be a good candidate to integrate vibrors regarding their qualities as non-invasive, low-powered, unobtrusive and small [10]. In addition, the socket already includes the surface electrodes that record the EMG signal. The space and location seems appropriate to contain both devices. However, we have to be sure that no interference will be produced between the vibrotactile stimulation and the myoelectric activity. Vibrotactile stimulation can also be used for different purposes: to feedback information of the action of the prosthesis, to prevent limitations such as maximum pinch strength, maximum opening or closing state, etc. Sensory substitution systems using vibrotactile stimulation can recreate information lost after an amputation. For example, it has been used to provide angular feedback of an elbow prosthesis and more recently for identifying level of grasping force [16, 47]. In both experiments, improvement in precision and accuracy were noticed for the users of the sensory substitution system. In addition, some manufacturers have already integrated vibration in their devices such as the I-limb®hand where vibration is used to inform the user when the hand is in a closed position.

Aside from vibrotactile stimulation, electrotactile stimulation is another interesting option that is actively investigated [1, 2, 11, 42]. Nevertheless, vibrations seems to be associated with higher participant preference, improvement in user performances and good compatibility for myoelectric prosthesis[23, 43, 48].

Using vibrotactile stimulation to substitute missing information might also have an impact on phantom limb pain (PLP) often described by patients suffering from an amputation [49, 50]. Theories about PLP highlighted that pain intensity might be related to the invasion of a neuron population of the body parts adjacent to the missing limb (maladaptive plasticity model), and/or the increased activity of the persistent representation of the missing limb (persistent representation model) [51, 52]. For both theories, the incongruent information between motor control signal and sensory feedback is a factor inducing pain [50, 53, 54]. Because sensory feedback stimulation such as mirror therapy and virtual reality have demonstrated positive effects on the reduction of phantom limb pain [55–58], we think that introducing a vibrotactile stimulus congruent with the motor intention/action as soon as possible after the amputation may contribute to limit maladaptive cortical plasticity, preserve correct limb representation and reduce or even prevent phantom limb pain development.

## Data Availability

The datasets used and/or analysed during the current study are available from the corresponding author on reasonable request.

## Abbreviations

CA: circular absolute arrangement
CP: circular proportional arrangement
I1, I2, I3: intensity of the stimulation (62.5, 100, 167mA) LA: linear absolute arrangement
LP: linear propoetional arrangement
OR: odd ratio
T1, T2, T3: duration of the stimulation (60, 100, 140ms)
