# Correction to: Effect of vibration characteristics and vibror arrangement on the tactile perception of the upper arm in healthy subjects and upper limb amputees

**DOI:** 10.1186/s12984-020-0656-z

**Published:** 2020-02-19

**Authors:** Matthieu Guemann, Sandra Bouvier, Christophe Halgand, Florent Paclet, Leo Borrini, Damien Ricard, Eric Lapeyre, Daniel Cattaert, Aymar de Rugy

**Affiliations:** 1 Team HYBRID; INCIA laboratory, CNRS UMR 5287, University of Bordeaux, 146 rue Leo Saignat, 33076 Bordeaux, France; 2 University Descartes, Paris, France; 3 Departement of Rehabilitation at the Army instruction Hospital, 1 Rue du Lieutenant Raoul Batany, 92190 Clamart, France; 4 Department of Neurology at the Army instruction Hospital, 1 Rue du Lieutenant Raoul Batany, 92190 Clamart, France; 5 Centre for sensorimotor performance HMNS, University of Queensland, Brisbane, Australia

**Correction to: J Neuroeng Rehabil**

10.1186/s12984-019-0597-6

The original article [1] contained an error whereby the captions to Fig. 3 and Fig. 8 were mistakenly interchanged. This has now been corrected.

**Fig. 3:**
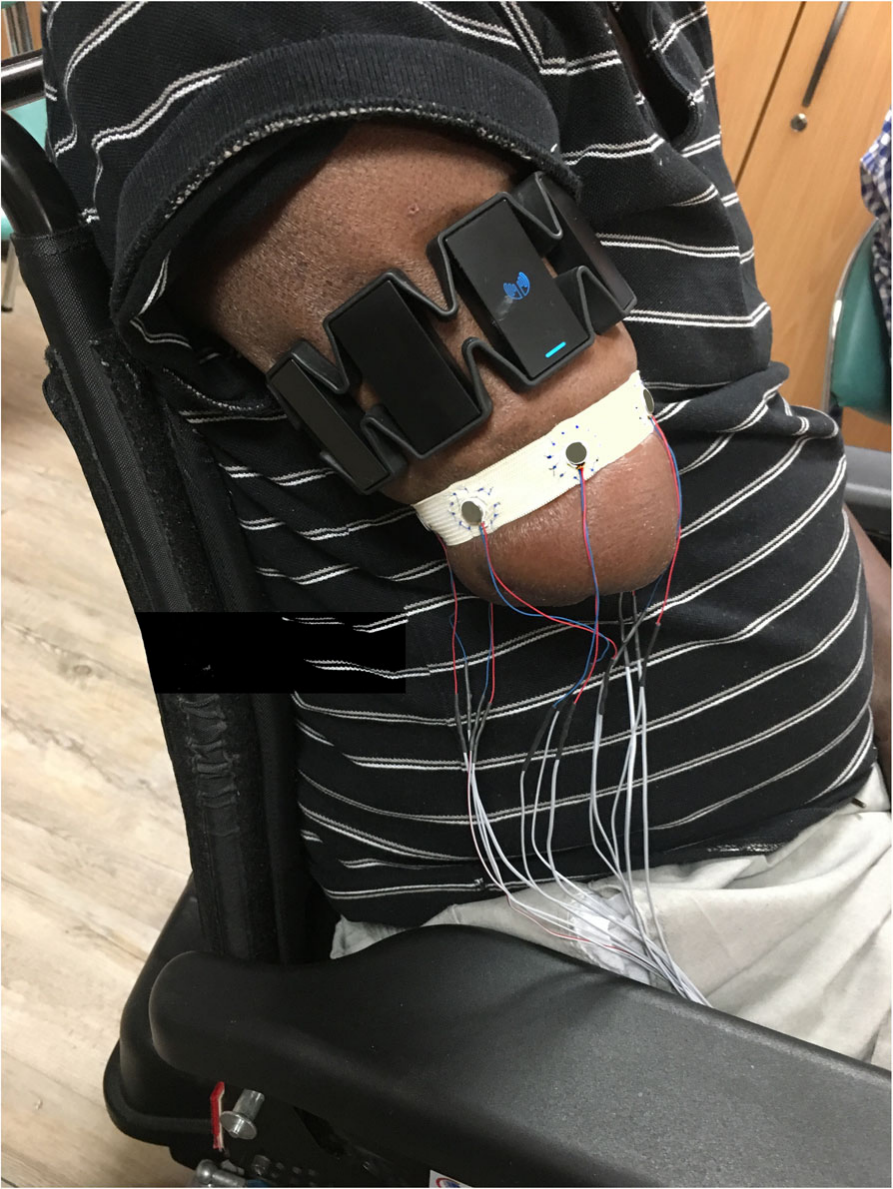
Example of one patient with vibrors encapsulated in a plastic piece and attached to an elastic band by surgical file

**Fig. 8:**
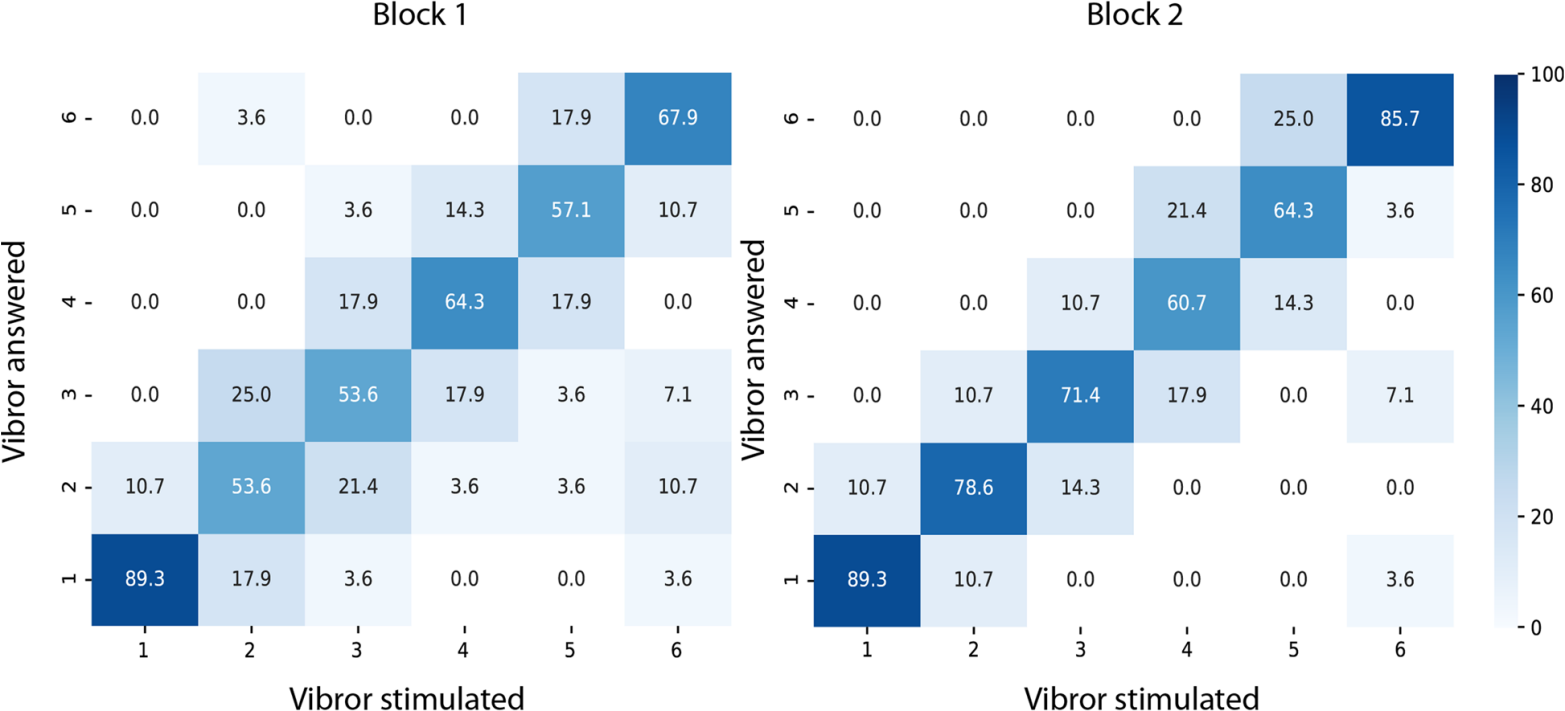
Confusion matrix representation of correct answers for the 7 patients testing the CP arrangement in the spatial discrimination task. Patients undewent two sessions. For both matrices, X axis represents the stimulation sent as “order” and the Y axis the patient’s answer. The gradient color corresponds to the recognition rate for each of these vibrors combinations. Darker color represents a higher recognition rate for the answered vibror. Correct answers correspond to the diagonal, for which the answered vibrors corresponded to the stimulated ones. Errors occurred whenever the answered number differed from the stimulated one

Furthermore, this error was mistakenly carried forward by the production department which handled this article, and thus was not the fault of the authors.
